# Comorbidities among the HIV-Infected Patients Aged 40 Years or Older in Taiwan

**DOI:** 10.1371/journal.pone.0104945

**Published:** 2014-08-13

**Authors:** Pei-Ying Wu, Mao-Yuan Chen, Szu-Min Hsieh, Hsin-Yun Sun, Mao-Song Tsai, Kuan-Yeh Lee, Wen-Chun Liu, Shan-Ping Yang, Yu-Zhen Luo, Jun-Yu Zhang, Wang-Huei Sheng, Chien-Ching Hung

**Affiliations:** 1 Center of Infection Control, National Taiwan University Hospital, Taipei, Taiwan; 2 Department of Internal Medicine, National Taiwan University Hospital and National Taiwan University College of Medicine, Taipei, Taiwan; 3 Department of Internal Medicine, Far Eastern Memorial Hospital, New Taipei City, Taiwan; 4 Department of Internal Medicine, National Taiwan University Hospital Hsin-Chu Branch, Hsin-Chu, Taiwan; 5 Department of Medical Research, China Medical University Hospital, Taichung, Taiwan; 6 China Medical University, Taichung, Taiwan; National Taiwan University Hospital, Taiwan

## Abstract

**Background:**

With the widespread use of combination antiretroviral therapy (cART), life expectancy of HIV-infected patients has significantly prolonged. An increasing number of HIV-infected patients are aging and concurrent use of medications are not uncommon for management of metabolic complications and cardiovascular diseases related to aging and prolonged exposure to cART.

**Methods:**

We reviewed medical records of all HIV-infected patients aged 40 years or older who had been followed at a university hospital for HIV care in Taiwan between January and December 2013. A standardized case record form was used to collect information on demographics and clinical characteristics, comorbidity, cART, and concurrent medications.

**Results:**

During the study period, 610 patients aged 40 to 49 years (mean, 44.1) and 310 aged 50 years or older (mean, 58.8) sought HIV care at this hospital. Compared with patients aged 40 to 49 years, those aged 50 years or older were significantly more likely to be female (15.9% vs 3.8%); to have received cART (97.7% vs 94.8%) and a lower plasma HIV RNA load (1.6 vs 1.7 log_10_ copies/ml); and to have diabetes mellitus (18.4% vs 4.6%), hypertension (31.0% vs 10.8%), hyperlipidemia (29.4% vs 11.6%), coronary artery disease (6.8% vs 0.5%), and an estimated glomerular filtration rate <60 ml/min/1.73 m^2^ (11.5% vs 2.7%); and were significantly less likely to have syphilis. Other than HIV infection, patients aged 50 years or older were more likely to have been receiving two or more concurrent medications than those aged 40 to 49 years (22.9% vs 6.4%).

**Conclusions:**

Our findings show a significant proportion of the HIV-infected patients aged 50 years or older have multiple comorbidities that may increase the risk for cardiovascular and renal complications. Issues of poly-pharmacy among the HIV-infected patients who are aging should be addressed to ensure adherence and minimize drug-drug interactions.

## Introduction

The widespread use of combination antiretroviral therapy (cART) has markedly improved the survival of HIV-1-infected patients [1], [2]. According to the multinational cohort study, the life expectancy of HIV-infected patients aged 20 years is projected to increase from 36.1 years to 49.4 years [1]. As a result of reduction in AIDS-related mortality, the number of elderly people living with HIV will continue to increase; furthermore, the number of cases of newly diagnosed HIV infection among the elderly people is increasing in many countries [3]–[5], which will have considerable impact on the future delivery of care in the developed as well as developing countries where cART coverage is increasing.

Prolonged exposure to antiretroviral therapy along with aging may increase risk of developing metabolic complications and cardiovascular diseases among HIV-infected patients. Several studies have provided evidence that comorbidities such as diabetes mellitus [6]–[11], hypertension [6], [8], [9], [12], coronary artery disease [11], hyperlipidemia [13], [14], renal disease [7], [11], [15], and reduced bone mineral density [16]–[18] are more common among HIV-infected elderly patients than HIV-uninfected controls. Other than cART, medications for management of metabolic complications and cardiovascular diseases will be needed, which will increase the pill burden and potential for drug-drug interactions in the elderly patients [19],[20].

The prevalence of HIV-1 infection continues to increase with improvement of accessibility to HIV testing and care in Taiwan [21], [22]. However, the information on comorbidities among the HIV-infected patients has been lacking. The purpose of this study aimed to describe the comorbidity profile and concurrent medications used among the elderly patients with HIV-1 infection who sought care at a referral medical center in Taiwan.

## Methods

### Study setting and population

After the first case of HIV-1 infection and AIDS was reported in Taiwan in 1984, the number of patients diagnosed as having HIV infection through sexual contacts continued to increase steadily over the next two decades [23]. The outbreak of HIV infection among injecting drug users (IDUs) between 2003 and 2007 had caused a significant change of the landscape of HIV infection in Taiwan, with the proportion of IDUs increasing from less than 2% before 2003 to 27.6% of total cases reported as of 2013. At the end of 2013, a total of 26,457 cases of HIV infection were reported to the Taiwan Centers for Disease Control and 4,171 (15.8%) patients had died. After control of the outbreak of HIV infection among IDUs through harm reduction program, sexual contacts, especially among men who have sex with men (MSM), have re-emerged as the most common route of HIV transmission. As of December 2013, MSM have accounted for 53% of all reported cases of HIV infection and patients aged 50 years or older accounted for 13.6% [21], [22].

HIV-infected patients in Taiwan are provided with free medical care at designated hospitals around Taiwan by the government of Taiwan, including cART that was introduced in April 1997, and monitoring of CD4 lymphocyte count and plasma HIV RNA load. As of December 2012, it was estimated that 60% of HIV-infected patients sought HIV-related care and initiated cART after the diagnosis of HIV infection was made.

### Study design

This was a cross-sectional study that enrolled HIV-infected patients who were aged 40 years or older and sought HIV care at the HIV clinics of the National Taiwan University Hospital from 1 January, 2013 to 31 December, 2013. Two age groups were identified: patients aged 40–49 years and those aged ≧50 years. We collected information on their baseline demographics, clinical characteristics, and medications including antiretroviral therapy, antihypertensives, lipid-lowering agents, and insulin and oral hypoglycemics. Data on comorbid conditions, including hypertension, diabetes mellitus, chronic viral hepatitis, cardiovascular disease, hyperlipidemia, fracture, malignancy, and osteoporosis and information on smoking status and alcohol use were also collected by interview, which were confirmed by review of medical records. The study was approved by the Research Ethics Committee of the National Taiwan University Hospital (registration number: 200904020R) and participants gave written informed consent.

### Clinical measurements

Systolic and diastolic blood pressure was measured after the subjects were seated and rested for at least five minutes. Height was determined without shoes by the same machine. Weight was measured by a digital scale, and patients were fully dressed but without shoes or heavy clothing. Body mass index (BMI) was calculated as weight in kilograms divided by the square of the height in meters. Because diabetes mellitus is considered coronary artery disease-equivalent, we examined the status of diabetes mellitus control by retrospectively collecting laboratory data of fasting glucose, glycosylated hemoglobin (HbA1C) among those who received a diagnosis of diabetes mellitus from 1 January, 2012 to 31 December, 2013.

### Laboratory and radiologic investigations

Total cholesterol, triglyceride, glucose, HbA1C, high-density lipoprotein cholesterol (HDL-C), and low-density lipoprotein-cholesterol (LDL-C) levels were determined after at least an 8-hour fast. The data of most recent plasma HIV RNA load, CD4 lymphocyte count, blood urea nitrogen, serum creatinine, estimated glomerular filtration rate (eGFR) that was calculated with the use of the abbreviated Modification of Diet in Renal Disease (MDRD) equation [24], hemoglobin, and rapid plasma reagin (RPR) (within 6 months of survey) were collected using a standardized case record form. Plasma HIV RNA load was quantified using the Cobas Amplicor HIV-1 Monitor test (Cobas Amplicor version 1.5, Roche Diagnostics Corporation, IN, USA) with a lower detection limit of 20 copies/mL, and CD4 count was determined using FACFlow (BD FACS Calibur, Becton Dickinson, CA, USA). Bone mineral density (BMD) was measured with the use of dual-energy X-ray absorptiometry scan (Lunar Prodigy; GE Healthcare, Belgium) [17].

### Definitions

Comorbid conditions were defined by the ICD-9 diagnostic codes or in those who took antihypertensives, hypoglycemics, or lipid-lowering agents. Chronic hepatitis B virus (HBV) infection was defined as presence of HBV surface antigen for 6 months or longer. Hepatitis C virus (HCV) infection was defined as presence of HCV antibody. Osteopenia and osteoporosis were defined on the basis of World Health Organization (WHO) criteria [25]. Patients with a BMD T-score between −1.0 and −2.5 were categorized as having osteopenia, and patients with a BMD T-score less than or equal to −2.5 were categorized as having osteoporosis.

CART was defined as the use of at least 3 agents from at least 2 classes of antiretroviral agents according to the national treatment guidelines for adults with HIV infection. The most commonly prescribed antiretroviral combinations in antiretroviral-naive patients were 2 nucleoside reverse-transcriptase inhibitors (NRTIs) plus 1 non-NRTI (nNRTI), 2 NRTIs plus ritonavir-boosted protease inhibitor (PI), unboosted atazanavir or raltegravir, while triple NRTIs or combination of 3 classes were only infrequently prescribed. Ritonavir was available only in capsule form.

### Statistical analysis

Categorical variables were analyzed by using X^2^ tests for continuous variables were compared using Student's *t* test. Baseline and comorbid conditions were compared with two groups. A *P*-value of <0.05 was considered statistically significant. All *P* value was two-tailed. Analyses were performed using SAS software (Version 9.3).

## Results

**Figure 1:**
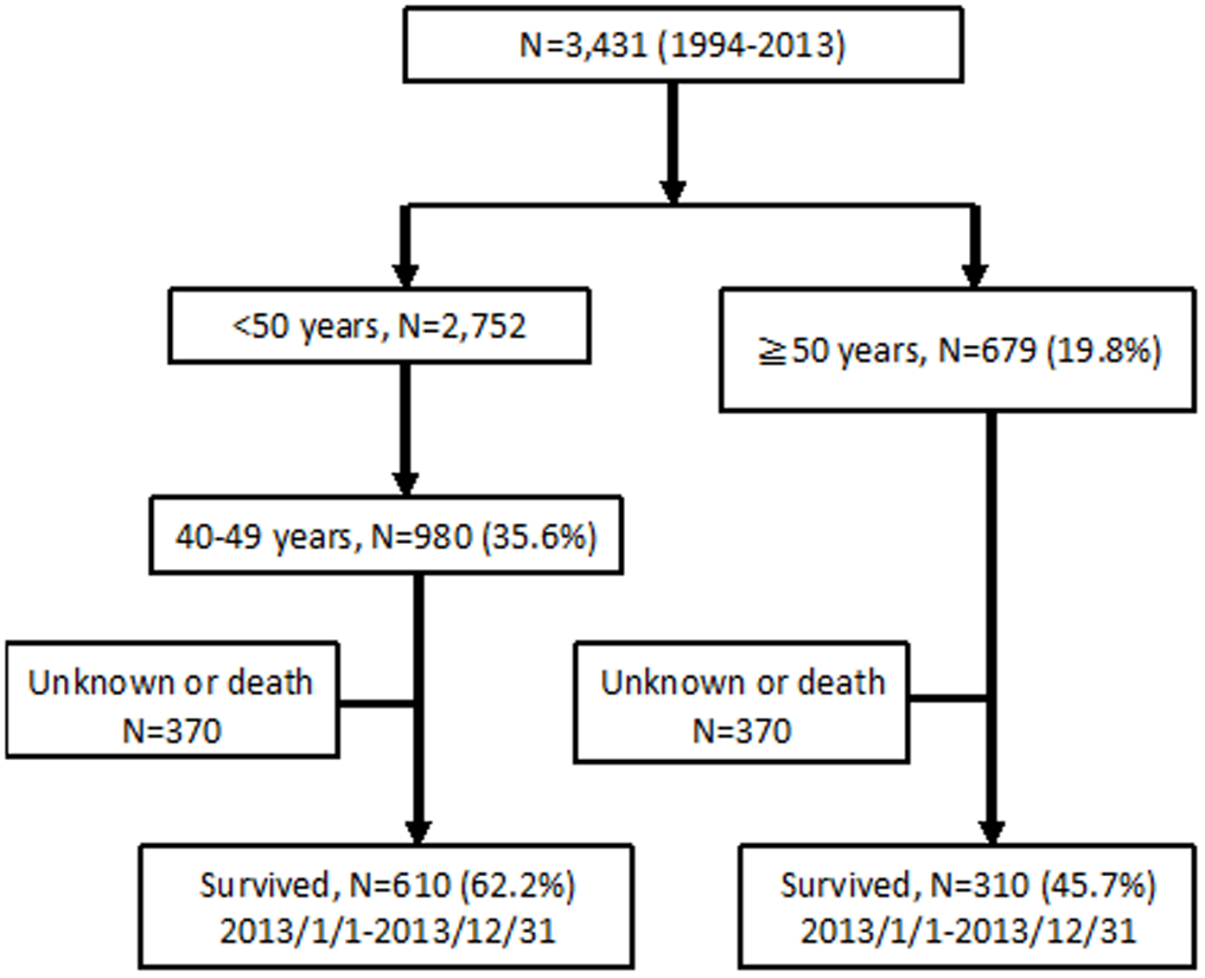
Study flow.

During the 12-month study period, 920 patients who were aged 40 years or greater were enrolled, among whom 310 (33.7%) and 610 (66.3%) were aged ≧50 years (elderly group) and 40–49 years (younger group), respectively (Figure 1). Clinical characteristics of the participants, stratified by age, are shown in Table 1. Of the 815 patients with available data, 304 (37.3%) were classified as overweight with a BMI >24 according to the criteria of the National Bureau of Health Promotion, Ministry of Health and Welfare, Taiwan; 31.0% (231/745) had fasting glucose levels ≧100 mg/dl, 51.5% (456/885) triglyceride levels ≧150 mg/dl, 20.0% (112/561) HbA1C levels≧6.0%, and 5.7% (49/854) eGFR levels <60 mL/min/1.73 m^2^.

**Table 1:** Comparisons of demographic and clinical characteristics of the two study populations according to age.

Variable	≧50 years, N = 310	40–49 years, N = 610	P
Male, n (%)	262 (84.5)	587 (96.2)	<.0001
Age, mean (SD), years	58.8 (7.8)	44.1 (2.9)	<.0001
Risk behavior, n (%)			<.0001
MSM	152 (49.0)	521 (85.4)	
Heterosexual	139 (44.8)	64 (10.5)	
IDU	12 (3.9)	22 (3.6)	
Other	7 (2.3)	3 (2.3)	
Smoking status, n (%) (N = 290, 550)			
Never	149 (51.4)	269 (48.9)	0.50
Past	64 (22.1)	80 (14.6)	<.01
Current	77 (26.6)	201 (36.6)	<.01
Body mass index, mean (SD), Kg/m^2^	22.9 (3.3)	23.4 (3.4)	0.02
(N = 287, 552)			
Systolic blood pressure, mean (SD), mm Hg (N = 286, 540)	130 (18.7)	126 (15.5)	<0.001
Diastolic blood pressure, mean (SD), mm Hg,	78 (11.9)	80 (11.4)	0.01
(N = 286, 540)			
Plasma HIV RNA load, mean (SD), log_10_ copies/ml (N = 306, 603)	1.6 (0.7)	1.7 (1.0)	0.006
CD4, mean (SD), cells/µl, (N = 308, 602)	525 (273)	552 (278)	0.2
TG, mean (SD), mg/dl (N = 300, 585)	199 (148.2)	203 (177.8)	0.73
TG≧150 mg/dl, n (%)	162 (54.0)	294 (50.3)	0.29
T-cholesterol, mean(SD), mg/dl (N = 300, 579)	180 (39.7)	178 (37.0)	0.34
T-cholesterol≧220 mg/dl, n (%)	54 (18.0)	75 (13.0)	0.05
HDL, mean (SD), mg/dl (N = 26, 87)	44 (15.2)	40 (10.1)	0.24
HDL<40 mg/dl, n (%)	12 (46.2)	46 (52.9)	0.55
LDL, mean (SD), mg/dl (N = 38, 74)	105 (40.7)	104 (31.9)	0.85
Fasting glucose, mean (SD), mg/dl (N = 261, 484)	106 (32.8)	97 (25.3)	<.001
Fasting glucose≧100 mg/dl, n (%)	116 (44.4)	115 (23.8)	<.0001
Fasting glucose≧110 mg/dl, n (%)	59 (22.6)	51 (10.5)	<.0001
HbA1C, mean (SD) (N = 215, 346)	5.9 (1.0)	5.6 (0.9)	<.0001
HbA1C≧6.5%, n (%)	32 (14.9)	18 (5.2)	<.0001
BUN, mean (SD) (N = 214, 395)	18.1 (13.2)	14.5 (6.7)	<.001
Serum creatinine, mean (SD) (N = 297, 553)	1.1 (1.2)	0.9 (0.8)	0.12
eGFR, mean (SD), ml/min/1.73 m^2^ (N = 297, 557)	88.5 (27.4)	98.9 (24.9)	<.0001
eGFR <60 ml/min/1.73 m^2^, n (%)	34 (11.5)	15 (2.7)	<.0001
On cART, n (%)	303 (97.7)	578 (94.8)	0.03
Current exposure to PI	185 (59.7)	325 (53.3)	0.06
Current exposure to TDF	92 (29.7)	246 (40.3)	0.002

**Note**: The numbers in the parenthesis presented after each variable indicate the number of patients with data for the elderly and younger groups of patients, respectively.

**Abbreviations**: BUN, blood urea nitrogen; cART, combination antiretroviral therapy; eGFR, estimated glomerular filtration rate; HbA1C, glycosylated hemoglobin, HDL, high-density lipoprotein cholesterol; IDU, injecting drug use; LDL, low-density lipoprotein cholesterol; MSM, men who have sex with men; PI, protease inhibitor; SD, standard deviation; TDF, tenofovir disoproxil fumarate; TG, triglyceride.

The two groups of patients differed significantly in many characteristics examined (Table 1). Compared with patients in the younger group, HIV-infected patients in the elderly group were more likely to be female (15.9% vs 3.8%) and have a higher fasting glucose level (106 vs 97 mg/dl), HbA1C level (5.9 vs 5.6%) and systolic blood pressure (130 vs 126 mmHg), and have a lower eGFR (88.5 vs 98.9 ml/min/1.73 m2), plasma HIV RNA load (1.6 vs 1.7 log_10_ copies/ml) and BMI (22.9 vs 23.4 Kg/m^2^) (Table 1). Compared with the younger group, there was a statistically significantly higher prevalence of comorbidities in the elderly patients (59.0 vs 36.4%) (Table 2), such as hypertension (31.0 vs 10.8%), hyperlipidemia (29.4 vs 11.6%), diabetes mellitus (18.4 vs 4.6%), and coronary artery disease (6.8 vs 0.5%); furthermore, HIV-infected patients in the elderly group were more likely to have 2 or more comorbidities (30.6 vs. 8.7%).

**Table 2:** Comorbid conditions of the two study populations.

Variable	≧50 years, N = 310 (%)	40–49 years, N = 610 (%)	P
At least one comorbidity, n (%)	183 (59.0)	222 (36.4)	<.0001
Diabetes mellitus	57 (18.4)	28 (4.6)	<.0001
Hypertension	96 (31.0)	66 (10.8)	<.0001
Anti-HCV-positivity	18 (5.8)	46 (7.6)	0.33
Chronic HBV infection	21 (6.8)	63 (10.3)	0.08
Hyperlidemia	91 (29.4)	71 (11.6)	<.0001
Cancer	12 (3.9)	13 (2.1)	0.12
CAD	21 (6.8)	3 (0.5)	<.0001
Fracture	3 (1.0)	1 (0.2)	0.08
Osteoporosis (N = 99, 99)	7 (7.1)	2 (2.0)	0.09
Active drinking (N = 288, 551)	13 (4.5)	18 (3.3)	0.36
RPR≧4, n (%)	31 (11.4)	148 (26.3)	<.0001
Concurrent medications, n (%)			
Lipid-lowering agent	60 (19.4)	42 (6.9)	<.0001
Hypoglycemic agent	51 (16.5)	25 (4.1)	<.0001
Anti-hypertensives	82 (26.5)	60 (9.8)	<.0001
Hypnotics	59 (19.0)	100 (16.4)	0.31

**Note**: The numbers in the parenthesis presented after each variable indicate the number of patients with data for the elderly and younger groups of patients, respectively.

**Abbreviations**: CAD, coronary artery disease; HBV, hepatitis B virus; HCV, hepatitis C virus; RPR, rapid plasma regain.

Of the 920 patients, 76 (8.3%) were taking oral hypoglycemic agents or receiving insulin replacement for control of diabetes mellitus, 102 (11.1%) lipid-lowering agents, 142 (15.4%) antihypertensives, and 159 (17.3%) hypnotics drugs (Table 2). More than 95% of the patients (95.8%) were receiving cART during the study period. The antiretroviral agents to which the patients had been exposed and the cumulative exposure duration for each antiretroviral agent are shown in Table 3. Patients in the elderly group had significantly longer exposure durations to PIs, nNRTIs, and NRTIs than those in the younger group (Table 3). The antiretroviral agents and exposure durations for patients with at least 1 metabolic, cardiovascular, renal or hepatic comorbidity and those without any comorbidity are shown in Table 4.

**Table 3:** The cumulative exposure durations of antiretroviral agents of the two study populations.

Drug class and duration	≧50 years, N = 310	40–49 years, N = 610	P
PI, mean (SD), months	64.40 (46.6)	56.1 (40.8)	0.02
Lopinavir/ritonavir	50.26 (37.9)	46.10 (37.1)	0.41
Atazanavir	45.94 (29.7)	43.96 (29.9)	0.51
Darunavir	24.43 (24.5)	17.21 (15.8)	0.22
Indinavir	23.56 (25.9)	21.21 (19.6)	0.59
Saquinavir	19.10 (19.5)	17.48 (13.7)	0.74
NRTI, mean (SD), months	103.0 (57.7)	78.04 (55.1)	<.0001
Zidovudine	68.88 (53.8)	58.70 (48.8)	0.03
d4T/ddI/ddC	36.79 (41.1)	33.87 (37.7)	0.52
Abacavir/lamivudine	59.08 (37.1)	50.54 (32.6)	0.006
Tenofovir	21.29 (9.8)	18.60 (9.4)	0.02
nNRTI, mean (SD), months	71.35 (56.8)	54.88 (52.0)	0.0003
Efavirenz	75.73 (55.7)	56.17 (51.9)	0.0002
Nevirapine	45.79 (54.5)	35.36 (45.8)	0.17
Integrase inhibitor, mean (SD), months	27.65 (23.2)	17.93 (11.8)	0.07

**Abbreviations**: d4T, stavudine; ddI, didanosine; ddC, deoxycytidine; NRTI, nucleoside reverse-transcriptase inhibitors; nNRTI, non-nucleoside reverse-transcriptase inhibitors; PI, protease inhibitor.

**Table 4:** The cumulative exposure durations of antiretroviral agents of the two study populations.

Drug class and duration	Presence of any comorbidity (+), N = 405	Without any comorbidity(-), N = 515	P
PI, mean(SD) months	64.67 (45.9)	54.52 (40.2)	0.004
Lopinavir/ritonavir	50.63 (34.7)	45.51(38.9)	0.29
Atazanavir	46.78 (30.3)	42.78 (29.2)	0.16
Darunavir	22.24 (20.0)	16.27 (17.2)	0.17
Indinavir	25.46 (27.9)	18.76 (14.8)	0.11
Saquinavir	19.46 (19.4)	16.19 (11.0)	0.45
NRTI, mean (SD), months	99.78 (59.1)	75.99 (53.4)	<.0001
Zidovudine	69.73 (52.4)	55.93 (48.5)	0.003
d4T/ddI/ddC	35.75 (40.6)	34.43 (37.6)	0.77
Abacavir/lamivudine	57.75 (36.5)	50.29 (32.7)	0.01
Tenofovir	20.07 (9.6)	18.83 (9.5)	0.23
nNRTI, mean (SD), months	71.27 (64.8)	51.90 (52.6)	<.0001
Efavirenz	73.36 (54.6)	54.0 (51.9)	0.0001
Nevirapine	45.33 (51.7)	34.50 (47.1)	0.15
Integrase inhibitor, mean (SD), months	24.97 (18.7)	16.93 (14.3)	0.06

**Note**: The comorbidities include hypertension, diabetes mellitus, hyperlipidemia, coronary artery disease, chronic kidney disease (eGFR<60 ml/min/1.73 m^2^), malignancy, osteoporosis, and chronic viral hepatitis.

**Abbreviations**: d4T, stavudine; ddI, didanosine; ddC, deoxycytidine; NRTI, nucleoside reverse-transcriptase inhibitors; nNRTI, non-nucleoside reverse-transcriptase inhibitors; PI, protease inhibitor.

Compared with those in the younger group, HIV-infected patients in the elderly group were more likely to take medications for management of comorbidities: oral hypoglycemic agents or insulin replacement (16.5% vs 4.1%), lipid-lowering agents (19.4% vs 6.9%), and antihypertensives (26.5% vs 9.8%) (Table 2). Other than therapy for HIV infection, patients in the elderly group were more likely to have receiving 2 or more concurrent medications than those in the younger group (22.9% vs 6.4%). Patients in the younger group had a higher prevalence of syphilis (RPR titer ≧4) than those in the elderly group (26.3% vs. 11.4%).

The comparisons of clinical characteristics of 85 patients (9.2%) with diabetes mellitus and 835 patients (90.8%) without diabetes mellitus are shown in Table S1. Compared with those patients without diabetes mellitus, the patients with diabetes mellitus were more likely to be aged 50 years or greater (67.1 vs 30.3%) and to have a higher BMI (24.2 vs 23.2 Kg/m^2^), higher systolic blood pressure (134 vs 127 mmHg), higher triglyceride level (260 vs 195 mg/dl), lower eGFR (82.7 vs 96.7 mL/min), and lower plasma HIV RNA load (1.5 vs 1.7 log_10_ copies/ml).

Because diabetes mellitus is related to long-term metabolic, cardiovascular and renal complications, we examined the status of control by assessing the fasting glucose and HbA1C data of the patients with diabetes mellitus. All of the available results of blood sampling in fasting state (n = 270) of 85 patients with diabetes mellitus over the 12-month follow-up were stratified into 4 groups (Figure S1): group 1, HbA1C>6.5% and fasting glucose>110 mg/dl (53.3%; 144/270); group 2, HbA1C>6.5% and fasting glucose<110 mg/dl (6.7%; 18/270); group 3, HbA1C<6.5% and fasting glucose>110 mg/dl (21.9%; 59/270) and group 4, HbA1C<6.5% and fasting glucose<110 mg/dl (18.1%; 49/270).

## Discussion

In this cross-sectional study, we found that HIV-1-infected Taiwanese patients who were aged 50 years or older had significantly more comorbidities than those who were aged 40–49 years. Polypharmacy was not uncommon in that more than 20% of the elderly patients were taking 2 or more concurrent medications in addition to cART for HIV infection.

The prevalence of the elderly group with 2 or more comorbidities is higher than younger group in our study (30.6 vs. 8.6%), which is similar to what have been observed in HIV-infected elderly patients with access to cART in many Western countries [6]–[9], [13]. The types of comorbid diseases in our study are also similar to those reported among the HIV-infected elderly patients in Western countries, such as hypertension [7], [9], [12], [13] and hyperlipidemia [13]. Previous studies comparing the prevalence of comorbidities between HIV-infected and HIV-uninfected individuals showed discrepant results, however. The prospective cross-sectional study by Onen et al showed that HIV-infected patients in the US had a higher prevalence of hypertension and hypertriglyceridemia than HIV-uninfected individuals [18], while the VACS study by Oursler et al showed that HIV-uninfected individuals in the US had a higher prevalence of hypertension and diabetes mellitus than HIV-infected patients [12].

With the increasing prevalence of comorbidities, it is not surprising that, other than antiretroviral therapy, use of concomitant medications for management of comorbid diseases were common in our patients. It is therefore important to note some of the negative consequences as a result of polypharmacy. The patients taking multiple drugs for many chronic medical conditions are at potentially increased risk of drug-drug interactions and adverse drug events [20], [26]. Consistent with findings reported in the literature (range, 82–96%) [20], [26], [27], 52.3% of our patients aged ≧50 years and 29.2% of the patients aged 40–49 years in this study were receiving 1 or more concurrent medications.

The prevalence and incidence of diabetes mellitus are increasing worldwide, especially in the elder patients regardless of the status of HIV infection [10], [28]–[30]. Presence of diabetes mellitus is associated with a higher mortality rate among HIV-uninfected patients [31]–[34] because of increased risk of cardiovascular diseases, nephropathy, retinopathy, and neuropathy [35], [36]. Whether the incidence of diabetes mellitus is higher in HIV-infected patients than HIV-uninfected individuals remains controversial. In the French study, Capeau et al found a markedly higher incidence of diabetes mellitus in HIV-infected than HIV-uninfected population (14.1 vs. 4-6/1000 person-years) [37]. In the Denmark study, Rasmussen et al found HIV-infected individuals did not have an increased risk of developing diabetes mellitus compared to HIV-uninfected population (3.70 vs. 3.87/1000 person-years) [38]. The discrepancy of incidence of DM in the published studies may be due to differences in demographic factors such as age, gender and race, exposure duration to cART and regimens of cART used [39], [40].

Using Taiwan National Health Insurance data, Jiang et al have found that the prevalence of DM among women and men aged 40 to 59 years increased from 4.63% to 5.47% and from 4.97% to 7.56%, respectively, between 2000 and 2009 [33]; the prevalence of DM among women and men aged 60 to 79 years increased from 17.17 to 21.97% and from 13.60 to 13.97%, respectively, between 2000 and 2009. In this study that was conducted in 2013, we found that the prevalence of DM in HIV-infected males (N = 749) and females (N = 41) aged 40–59 years was 7.48% and 7.32%, respectively; for the patients who were aged 60 years or older (N = 130), the prevalence of DM in males (N = 100) and females (N = 30) was 21.0% and 16.67%, respectively. However, given the small sample size of the patients in our study, the interpretation of the data should be cautious.

In our study, we found that diabetes mellitus was associated with older age and higher BMI, which are consistent with other studies [10], [28], [29]. The finding that more than 50% of the blood sampling among HIV-infected patients with diabetes mellitus showed HbA1C >6.5% and fasting glucose levels >110 mg/dl suggests that better strategies with multidisciplinary approach are urgently needed to improve the quality of care in terms of diabetic control to prevent the occurrence or delay the progression of metabolic, cardiovascular, and renal complications in the patients enrolled in this study.

Renal function in terms of eGFR usually decreases with age. The prevalence of eGFR<60 mL/min/1.73 m^2^ in our elderly patients was 11.5%, which is higher than those in other studies among the HIV-infected population from Brazil (3.9%) [8] and Taiwan (7.03%) [41], but is lower than the studies from the UK (15.5%) [15] and Japan (15.4%) [42]. The difference between ours and other studies may be explained by the study populations. In our study population, the mean age was 49 years, which is greater than the other study from Taiwan (36.9 years). In the US study, Onen et al. found approximately 50% of the overall elderly study population had renal impairment with chronic kidney disease in 11% and 7% of HIV-infected persons and HIV-uninfected patients, respectively [18].

While most of patients in this study were receiving cART with good control of HIV infection, the finding of a high prevalence of syphilis is of particular concerns, especially in the patients aged 40 to 49 years [43], [44]. Acquisition of syphilis indicates unprotected sex, which may increase the risk of HIV transmission to sexual partners or superinfection with HIV resistant to the regimens the patients were receiving; furthermore, several studies have demonstrated that syphilis is associated with acquisition of other sexually transmitted hepatotrophic virus infections such as hepatitis B, C and D virus [45]–[48].

There are several limitations of our study. First, it is a cross-sectional survey of patients who sought HIV care at a university hospital. The information examined is mainly from laboratory data that were accumulated during the clinical care. Many other comorbidities were not systematically examined, such as osteoporosis and malignancy. Second, the exposure duration to cART was not taken into consideration in comparisons made between the two age groups. Third, we did not have an HIV-uninfected population for comparison in terms of the frequency of comorbidity. Therefore, it is not known whether the frequency of any comorbidity examined in this study is higher in HIV-infected patients than in HIV-uninfected patients in Taiwan, although several studies have suggested that cART and HIV infection may accelerate aging and increase risk of metabolic, cardiovascular and renal complications [10], [14], [15], [39], [40], [42].

In conclusion, our findings show that a significant proportion of the HIV-infected elderly patients In Taiwan have multiple comorbidities that may increase risk for cardiovascular and renal complications. Issues of poly-pharmacy among the elderly with HIV infection should be addressed to ensure adherence and minimize drug-drug interactions. Comprehensive approach to the management of metabolic, cardiovascular and renal comorbidities cannot be overemphasized in the long-term successful management of HIV-infected elderly population.

## Supporting Information

Figure S1 The scattered plot of glycosylated hemoglobin (HbA1C) and fasting glucose data collected from the 85 patients with diabetes mellitus during the 12-month study period. PI, protease inhibitor-containing regimens.
(TIF)

Table S1 Comparisons of demographic and clinical characteristics of the patients with and those without diabetes mellitus.
(DOCX)
