# Supplementary figures and images for: Comorbidities among the HIV-Infected Patients Aged 40 Years or Older in Taiwan
Source: PLoS One. 2014 Aug 13;9(8):e104945. doi: 10.1371/journal.pone.0104945 (PMC4132082; doi:10.1371/journal.pone.0104945)

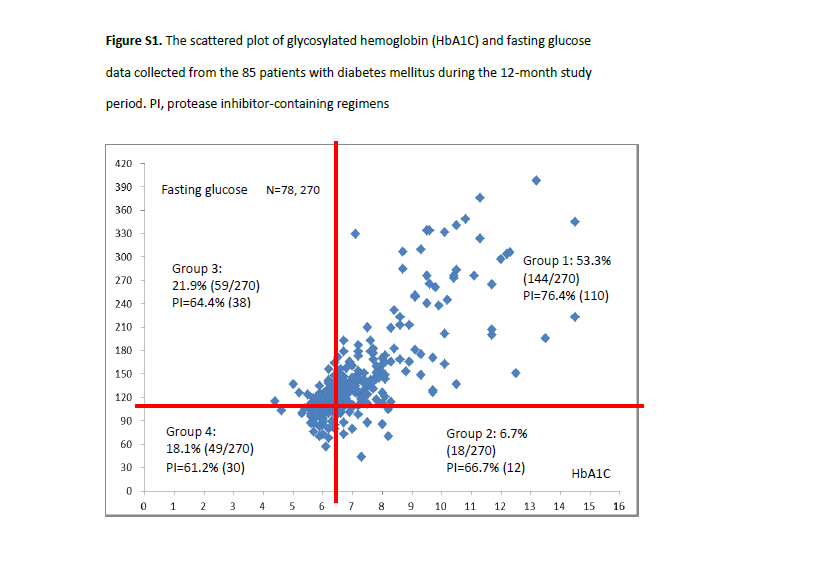

Supplement: Figure S1 — The scattered plot of glycosylated hemoglobin (HbA1C) and fasting glucose data collected from the 85 patients with diabetes mellitus during the 12-month study period. PI, protease inhibitor-containing regimens. (TIF) [file pone.0104945.s001.tif]
