# Supplementary material for: Comorbidities among the HIV-Infected Patients Aged 40 Years or Older in Taiwan
Source: PLoS One. 2014 Aug 13;9(8):e104945. doi: 10.1371/journal.pone.0104945 (PMC4132082; doi:10.1371/journal.pone.0104945)
Supplement: Table S1 — Comparisons of demographic and clinical characteristics of the patients with and those without diabetes mellitus. (DOCX) [file pone.0104945.s002.docx]

**Table S1.** Comparisons of demographic and clinical characteristics of the patients with and those without diabetes mellitus

| Variable | DM (+), | DM(-), | P |
| --- | --- | --- | --- |
|  |  |  |  |
| Male sex, n (%) | 77 (90.6) | 772 (92.4) | 0.54 |
| Age, mean (SD), years | 55.6 (9.8) | 48.4 (8.2) | <.0001 |
| Smoking status, n (%) (N=80, 760) |  |  |  |
| Never | 45 (56.3) | 373 (48.8) | 0.22 |
| Past | 19 (23.8) | 125 (16.5) | 0.09 |
| Current | 16 (20.0) | 262 (34.5) | <.01 |
| Active drinking, n (%) (N=79, 760) | 0 (0.00) | 31 (4.2) | 0.07 |
| Body mass index, mean (SD), kg/m^2^ (N=79, 760) | 24.2 (3.4) | 23.2 (3.3) | 0.01 |
| Systolic blood pressure, mean (SD), mm Hg  (N=79, 747) | 134 (19.0) | 127 (16.4) | <.001 |
| Diastolic blood pressure, mean (SD), mm Hg | 80 (11.2) | 79 (11.7) | 0.85 |
| (N=79, 747) |  |  |  |
| Plasma HIV RNA load, mean (SD), log_10_ copies/ml (N=83, 826) | 1.5 (0.8) | 1.7 (0.9) | 0.04 |
| CD4, mean (SD), cells/µl (N=83, 827) | 565 (248.7) | 541 (278.9) | 0.44 |
| TG, mean (SD), mg/dl (N=82, 803) | 260 (179.1) | 195 (166.1) | <.001 |
| TG≧150 mg/dl, n (%) | 61 (74.4) | 395 (49.2) | <.0001 |
| T-cholesterol, mean (SD), mg/dl (N=79, 800) | 169 (34.2) | 178 (38.2) | 0.02 |
| T-cholesterol≧220 mg/dl, n (%) | 7 (8.9) | 122 (15.3) | 0.13 |
| HDL, mean (SD), mg/dl (N=8, 105) | 34 (10.8) | 42 (11.4) | 0.06 |
| HDL<40 mg/dl, n (%) | 6 (75.0) | 52 (49.5) | 0.16 |
| LDL, mean (SD), mg/dl (N=18, 94) | 87 (30.3) | 108 (34.9) | 0.02 |
| LDL>130 mg/dl, n (%) | 1 (5.6) | 24 (25.5) | 0.06 |
| Fasting glucose, mean (SD), mg/dl (N=76, 669) | 148 (61.1) | 95 (14.2) | <.0001 |
| Fasting glucose≧100 mg/dl | 68 (89.5) | 163 (24.4) | <.0001 |
| Fasting glucose≧110 mg/dl | 59 (77.6) | 51 (7.6) | <.0001 |
| HbA1c, mean (SD) (N=74, 487) | 7.1 (1.5) | 5.6 (0.5) | <.0001 |
| HbA1c ≧6.5%, n (%) | 41 (55.4) | 9 (1.9) | <.0001 |
| Serum creatinine, mean (SD) (N=83, 767) | 1.5 (1.9) | 1.0 (0.8) | 0.02 |
| eGFR, mean (SD) (N=82, 772) | 82.7 (34.7) | 96.7 (24.8) | <.001 |
| eGFR <60 ml/min/1.73m^2^, n (%) | 20 (24.4) | 29 (3.8) | <.0001 |
| On cART, n (%) (N=85, 835) | 84 (98.8) | 797 (95.5) | 0.14 |
| RPR≧4, n (%) (N=72, 764) | 4 (5.6) | 175 (22.9) | <.001 |

**Note:** The two numbers shown in the parenthesis after each variable indicate the number of patients with data for the patients with DM and those without DM, respectively**.**

**Abbreviations:** BUN, blood urea nitrogen; cART, combination antiretroviral therapy; DM, diabetes mellitus; eGFR, estimated glomerular filtration rate; HbA1C, glycosylated hemoglobin, HDL, high-density lipoprotein cholesterol; LDL, low-density lipoprotein cholesterol; RPR, rapid plasma reagin; SD, standard deviation; TG, triglyceride

HbA1C

HbA1C

HbA1C

HbA1C

HbA1C

HbA1C

HbA1C

HbA1C
